# Comparative Analysis of Frailty Indices on Complication Risk Following Septic Revision Total Hip and Knee Arthroplasty

**DOI:** 10.1177/21514593251392692

**Published:** 2025-10-25

**Authors:** Hannah Grimmett, Arsalaan Sayyed, Victor Koltenyuk, Aruni Areti, Hikmat R. Chmait, Nithin Gupta, Mitchell Gray, William Young, Tyler K. Williamson, Chance Moore, Frank A. Buttacavoli

**Affiliations:** 1 472570 William Carey University College of Osteopathic Medicine, Hattiesburg, MS, USA; 2 REAM Orthopedics, Columbus, OH, USA; 3 364432 Campbell University School of Osteopathic Medicine, Lillington, NC, USA; 4 School of Medicine, New York Medical College, Valhalla, NY, USA; 5 3989 Baylor College of Medicine, Houston, TX, USA; 6 Larner College of Medicine, University of Vermont, Burlington, VT, USA; 7 Bowers Neurosurgical Frailty and Outcomes Data Science Lab, Flint, MI, USA; 8 Department of Orthopaedic Surgery, University of Vermont, Burlington, VT, USA; 9 Department of Orthopaedic Surgery, University of Texas Health San Antonio, San Antonio, TX, USA

**Keywords:** total joint arthroplasty, revision, frailty, mortality, complications, total hip arthroplasty

## Abstract

**Background:**

Frailty is an established risk factor for adverse outcomes following total joint arthroplasty, including higher rates of prosthetic joint infection (PJI), reoperation rates, and readmission, which may be greater in the setting of revision. The purpose of this study is to compare the association of frailty indices with mortality and complications following septic revision arthroplasty.

**Methods:**

A query from The American College of Surgeons National Surgical Quality Improvement Program (NSQIP) was performed for adult patients undergoing revision total knee or hip arthroplasty between 2015 and 2020, which records perioperative data (30 days postoperatively) for over 700 centers nationwide. PJI cases without revision arthroplasty were excluded. The RAI-rev and mFI-5 frailty scores were calculated for each patient. Outcomes included major complications, mortality, non-home discharge (NHD), DVT, readmission within 30 days, wound complications, pulmonary complications, cardiac complications, and postoperative infection. T-test and binary logistic regression assessed associations with frailty scores and outcomes. Predictability was evaluated through multivariate regression analysis, and its discriminative accuracy was measured using receiver operating curve (ROC) analysis and C-statistics.

**Results:**

A total of 4395 patients were included (median age: 66 [IQR 59-73]). Within the cohort, 46.44% were female and 38.02% exhibited NHD. RAI-rev demonstrated increased association compared to mFI-5 with mortality (OR: 1.20 vs 1.10, CI: 95%) and NHD (OR: 1.15 vs 1.05, CI: 95%). RAI-Rev demonstrated significantly superior discriminatory accuracy when compared to mFI-5 for NHD (Cs: 0.670 vs 0.602, *P* < 0.001) and mortality (Cs: 0.795 vs 0.574, *P* < 0.001).

**Conclusions:**

Frailty may have a distinct association with mortality and NHD following septic rTJA, especially when assessed by the revised Risk Analysis Index. This understanding is important to educate the patient and their family and provide insight into the necessary resources and surveillance needed to manage frail patients undergoing septic revision total joint arthroplasty.

## Introduction

As the United States population ages, the utilization of total hip (THA) and total knee arthroplasty (TKA) has continued to rise over the past two decades.^1,2^ Through 2060, the rate of THAs is projected to increase by 659% and TKAs by 469%.^1^ Although these surgical interventions provide numerous benefits to patients’ overall quality of life, 10% suffer from implant-associated complications with 1% of those being periprosthetic joint infections (PJI).^3^ PJI is a dreaded complication of joint arthroplasty, contributing to more than 1.65 billion healthcare dollars spent and a dramatic reduction in quality of life.^4^ The incidence of PJI has been estimated to be 1%–3% of TKAs and in 0.1%–1% of THAs. This incidence, according to recent reviews, continues to rise, with most resulting in the need for revision surgery with the revision of the TKA/THA potentially being associated with a higher mortality rate.^5-9^ Septic revision total joint arthroplasty (rTJA) has been proven to have a mortality rate of 33% compared to 11% for aseptic indications.^10^ While age has been noted as a risk factor for complications following rTJA, more comprehensive indices of current physiologic state, like frailty, may play a greater role.^5,11-13^

As a result of the increased mortality rate associated with septic rTJA, it is vital to utilize preoperative tools that accurately project risk for complications in the morbid, invasive setting of septic rTJA.^14^ Frailty, which can be defined as a decrease in physiologic reserve, in conjunction with other comorbidities, synergistically increases a patient’s risk of developing PJI following primary TJA.^15^ Thus, those developing PJI and undergoing rTJA may already be at increased risk for complications due to their frail physiologic profiles.^16,17^ Various indices, including the 5-factor modified frailty index (mFI-5) and the revised Risk Analysis Index (RAI-rev), have been developed and are used in the preoperative risk stratification of patients. Yet, no definitive frailty index has been designated as superior in predicting these adverse outcomes in this setting.^18-20^

As a result, surgeons have a limited capacity for preoperative optimization and risk stratification with respect to the utilization of frailty indices.^21^ Because of this gap in the literature, our objective is to help determine a gold standard frailty index by comparing the associations of the RAI-rev and the mFI-5 with 30-day mortality in patients undergoing septic rTJA, in addition to other relevant outcomes, including non-home discharge (NHD), major medical complications, and readmission. We hypothesize that RAI-rev will be a better predictor of adverse outcomes in patients following septic rTJA due to its more comprehensive domains in fully capturing a patient’s physiologic state.

## Methods

### Data Source and Patient Selection Criteria

We utilized the American College of Surgeons National Surgical Quality Improvement Program (NSQIP) from the years 2015-2020. The NSQIP is a national database with risk-adjusted surgical outcomes data in the 30-day postoperative period.^22^ Patients included in this study were identified using *The Current Procedural Terminology* (CPT) codes 27 091, 27 130, and 27 134 for revision THA and 27 447, 27 487, and 27 488 for revision TKA. The 10th revision of *The International Classification of Diseases* (ICD-10) codes for infection and inflammatory reaction due to internal hip (T84.51XA and T84.52XA) or knee prosthesis (T84.53XA and T84.54XA) and the 9th revision of *The International Classification of Diseases* (ICD-9) code for infection and inflammatory reaction due to internal prosthetic joint prosthesis (996.66) were applied to include patients undergoing septic revision of either THA or TKA. Patients were excluded if undergoing concomitant procedures.

### Calculation of Frailty Using mFI-5 and RAI

The mFI-5 was developed from the original 11-item Modified Frailty Index (mFI-11) to accommodate the removal of 6 variables from the NSQIP which were previously used to calculate the mFI-11.^23^ The mFI-5 was calculated by assigning 1 point to each of the 5 variables if present in a patient **(**Supplemental Table S1**)**. The following cutoffs were then used to group patients by frailty status as measured by the mFI-5: Non Frail (mFI-5 = 0), Prefrail (mFI-5 = 1), Frail (mFI-5 = 2), and Severely frail (mFI-5 ≥3).

The RAI was originally developed by Hall et al^19^ as a more comprehensive measure of frailty compared to existing frailty indices, as it utilizes 11 weighted variables that encompass multiple domains of frailty **(**Supplemental Table S2**)**. It was then recalibrated for use in surgical patients using the Veterans Affairs Surgical Quality Improvement Program and externally validated using a prospective cohort.^24^ Patients are scored on a scale from 0-81, with the 90th percentile of RAI scores representing severely frail patients. The RAI frailty tiers used in this study were Robust (RAI ≤15), Normal (RAI 16-25), Frail (26-35), and Severely frail (RAI ≥36; Supplemental Table 3).^24^

### Demographic and Clinical Characteristics

Descriptive variables regarding demographic and clinical characteristics were collected for the total cohort and for each tiered frailty category. Demographic variables included age, sex, race, Hispanic ethnicity, body mass index (BMI), and obesity class. Preoperative variables included American Society of Anesthesiologists (ASA) score, hypertension requiring medication, diabetes requiring oral medication or insulin, history of severe chronic obstructive pulmonary disease (COPD), diagnosis of congestive heart failure, functional health status (independent, partially or totally dependent), and diagnosis of cancer. Intraoperative and postoperative variables included operative time, length of hospital stay, NHD, and 30-day complications such as readmission, wound complications, and mortality. Continuous variables were reported as median values with interquartile range (IQR). Preoperative comorbidities and 30-day outcome variables were reported as counts with percentages.

### Primary and Secondary Endpoints

The primary endpoint for this study was mortality within 30 days following septic revision THA or TKA. A sub-analysis was performed to investigate secondary endpoints including the following: non-home discharge (NHD), major complications (those requiring an ICU admission or surgical intervention to address), minor complications, pulmonary complications, cardiac complications, wound complications, deep vein thrombosis (DVT), readmission within 30 days, and postoperative infection (including superficial SSI, deep incisional SSI, and organ space SSI).

### Statistical Analysis

Multivariate analysis was performed controlling for age and sex for frailty as a predictor of the primary and secondary endpoints comparing mFI-5 and RAI-rev. Results of multivariate logistic regression are represented with an odds ratio (OR) with 95% confidence interval (95% CI) and a c-statistic (Cs) with 95% confidence interval (95% CI). The discriminatory accuracy of each frailty model was assessed using Receiver Operating Characteristic (ROC) analysis and quantified with the area under the curve/C-statistic. The discriminatory accuracy of mFI-5 and RAI-rev for each outcome was compared using the DeLong test. Statistical significance was indicated by *P*-value <0.05. All statistical analysis was performed using Statistical Package for Social Sciences (IBM, SPSS Statistics, Armonk, NY).

## Results

### Cohort Characteristics

The total patient cohort consisted of 4395 patients who met the inclusion criteria for this study (Table 1). The median age of the cohort was 66 years (IQR 59-73), with females comprising 46.4% of the population. Among the cohort, 1671 (38.0%) experienced NHD, and 41 (0.9%) suffered from mortality. Additionally, 214 (4.9%) patients experienced major complications and 404 (9.2%) suffered from minor complications. Outcomes were assessed by sex and age groups (Tables 2 and 3). When examining frailty status, the largest subset of patients by RAI-Rev was in the Frail group, representing 2409 (54.8%) of the cohort. This was followed by the RAI-Rev Prefrail group at 1608 (36.6%), and the Severely Frail group at 303 (6.9%). In contrast, the mFI-5 classification showed that 2034 (46.3%) patients were Prefrail, while 981 (22.3%) were categorized as Frail and 111 (2.5%) as Severely Frail.

**Table 1.** Baseline Demographic Variables, Clinical Characteristics, and Postoperative Outcomes Including Incidence of 30-Day Mortality, Readmission, Major Complications, and Discharge Disposition of Patients Undergoing a Revision of a Septic Total Hip or Total Knee Arthroplasty

Variable	Cohort (n = 4395)
**Age** (median, IQR)	66 years (59-73)
**Female patients** (n,%)	2041 (46.4)
**BMI** (median, IQR)	32.1 kg/m^2^ (29.5-34.1)
**Length of stay** (median, IQR)	4 days (2-6)
**Operative time** (median, IQR)	147 min (106-188)
**Mortality** (n, %)	41 (0.9)
**Readmission** (n, %)	468 (10.6)
**Major complications** (n, %)	214 (4.9)
**Minor complications** (n, %)	404 (9.2)
**Septic joint location** (n, %)	
Hip	1959 (29.0)
Knee	4812 (71.3)
**Discharge destination** (n, %)	
Home	2724 (62.0)
Non-routine (rehab, SNF, and others)	1671 (38.0)
**Functional health status** (n, %)	
Independent	4049 (92.1)
Partially dependent	276 (6.3)
Totally dependent	24 (0.5)
**Preoperative clinical status/Comorbidities** (n, %)	
Diabetes mellitus	1083 (24.6)
Severe COPD	271 (6.2)
CHF	52 (1.2)
Current smoker	633 (14.4)
Disseminated cancer	22 (0.5)
Open wound/Wound infection	386 (8.8)
Ventilator dependent	7 (0.2)
**Frailty distributed based on RAI-rev** (n, %)	
Robust	75 (1.7)
Prefrail	1608 (36.6)
Frail	2409 (54.8)
Severely frail	303 (6.9)
**Frailty distributed based on mFI-5** (n, %)	
Robust	1269 (28.9)
Prefrail	2034 (46.3)
Frail	981 (22.3)
Severely frail	111 (2.5)

**Table 2.** ANOVA for Each Age Category for Each Primary Outcome

Age groups	<55	56-65	66-75	>75	*P*-value (two-sided)
Mortality	0.3%	0.4%	0.7%	3.0%	<.001
Major complications	7.6%	10.0%	8.8%	10.0%	.239
Non-home discharge	21.0%	30.1%	42.1%	60.6%	<.001
Wound complications	4.0%	3.1%	3.2%	2.9%	.604
DVT	1.7%	1.0%	1.2%	1.0%	.557
Readmission within 30 Days	8.9%	12.2%	10.0%	10.8%	.086
Pulmonary complications	1.3%	1.3%	2.0%	2.7%	.087
Cardiac complications	0.4%	0.5%	0.8%	1.1%	.245
Infection	7.1%	8.6%	8.9%	9.4%	.401

**Table 3.** ANOVA for Sex for Each Primary Outcome

Sex	Maie	Female	*P*-value (two-sided)
Mortality	0.7%	1.2%	.119
Major complications	9.8%	8.5%	.126
Non-home discharge	33.7%	43.0%	<.001
Wound complications	3.6%	2.8%	.152
DVT	1.5%	0.8%	.035
Readmission within 30 Days	10.7%	10.6%	.896
Pulmonary complications	2.0%	1.6%	.286
Cardiac complications	0.8%	0.6%	.614
Infection	8.2%	9.0%	.335

### Mortality after Septic Revision

**Figure 1.**
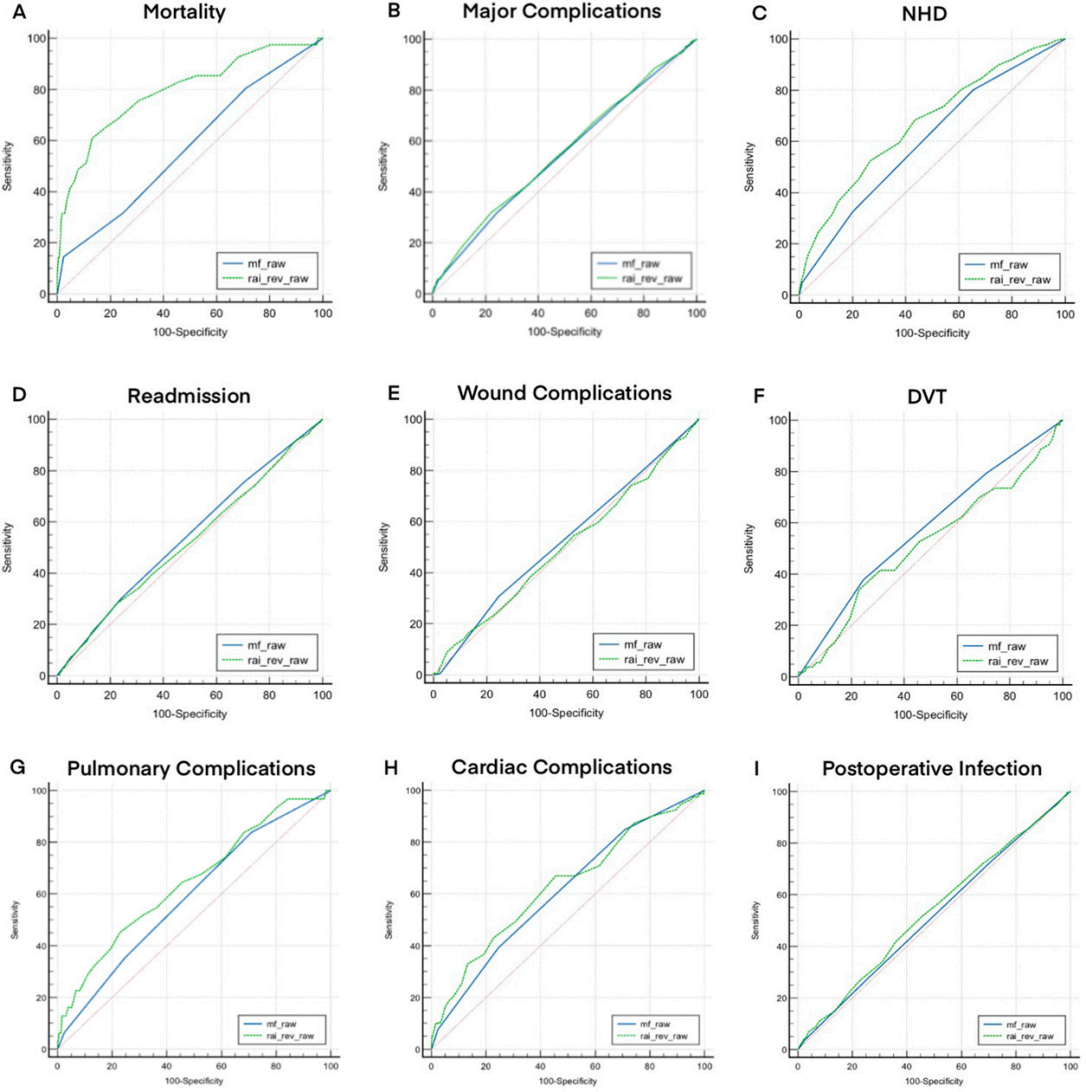
AUROC Analysis Quantifying the Discriminatory Accuracy of Frailty as Measured by the RAI and mFI-5 for Mortality (A), Major Complications (B), Non-home Discharge (C), Readmission Within 30 days (D), Wound Complications (E), DVT (F), Pulmonary Complications (G), Cardiac Complications (H), and Postoperative Infection (I)

After controlling for age and sex, a multivariate analysis was conducted to assess associations of frailty indices with mortality for each cohort (Figure 1, Tables 4 and 5). With each increasing point in RAI-rev, the odds of mortality (OR: 1.20, 95% CI: 1.14-1.25) significantly increased, as it also did with mFI-5 (OR: 1.10, 95% CI: 1.06-1.14; Table 5).

**Table 4.** Multivariate Logistic Regression Controlling for Age and Sex for Each Frailty Category for Each Primary Outcome. Results Are Presented as Odds Ratio (OR) and 95% Confidence Interval

	Odds (95% confidence interval)					
	RAI-rev					
	Prefrail	*P*-value	Frail	*P*-value	Severely frail	*P*-value (two-sided)
Mortality	n/a	n/a	1.02 (0.31-3.31)	0.973	5.64 (1.41-22.60)	0.015
Major complications	3.06 (0.91-10.32)	0.072	4.57 (1.26-16.63)	0.021	8.29 (2.11-32.59)	0.002
Non-home discharge	2.00 (0.84-4.79)	0.12	2.81 (1.24-7.03)	0.027	9.39 (3.55-24.87)	<0.001
Wound complications	1.09 (0.29-4.01)	0.903	1.00 (0.22-4.47)	0.998	1.88 (0.35-10.04)	0.461
DVT	1.25 (0.13-11.60)	0.847	1.43 (0.11-18.12)	0.784	1.43 (0.08-26.59)	0.811
Readmission within 30 Days	1.12 (0.50-2.54)	0.782	1.29 (0.51-3.21)	0.592	1.70 (0.61-4.72)	0.311
Pulmonary complications	0.78 (0.09-6.71)	0.822	0.78 (0.70-8.20)	0.834	2.18 (0.18-27.19)	0.545
Cardiac complications	n/a	n/a	1.06 (0.33-3.42)	0.928	3.33 (0.71-15.67)	0.127
Infection	1.45 (0.54-3.89)	0.464	2.02 (0.68-6.00)	0.203	2.76 (0.84-9.07)	0.095
	**mFI-5**					
	**Prefrail**	***P*-value**	**Frail**	***P*-value**	**Severely frail**	***P*-value** (two-sided)
Mortality	1.12 (0.49-2.58)	0.791	0.78 (0.28-2.18)	0.628	6.11 (2.02-18.46)	0.001
Major complications	1.16 (0.89-1.51)	0.283	1.50 (1.11-2.02)	0.008	3.16 (1.88-5.33)	<0.001
Non-home discharge	1.43 (1.22-1.69)	<0.001	1.98 (1.64-2.39)	<0.001	6.04 (3.85-9.49)	<0.001
Wound complications	1.05 (0.68-1.60)	0.838	1.59 (1.00-2.52)	0.05	0.32 (0.34- 2.39)	0.269
DVT	1.37 (0.64-2.92)	0.417	2.32 (1.05-5.13)	0.037	2.32 (0.50-10.87)	0.286
Readmission within 30 Days	1.23 (0.96-1.58)	0.098	1.55 (1.17-2.05)	0.002	1.81 (1.02-3.20)	0.041
Pulmonary complications	1.71 (0.87-3.33)	0.118	2.42 (1.19-4.92)	0.014	5.23 (1.90-14.41)	0.001
Cardiac complications	1.59 (0.57-4.45)	0.377	1.93 (0.63-5.88)	0.249	3.72 (0.71-19.74)	0.123
Infection	1.05 (0.81-1.37)	0.711	1.09 (0.80-1.48)	0.596	1.47 (0.79-2.73)	0.221

**Table 5.** Multivariate Logistic Regression Controlling for Age and Sex Across Continuous Frailty Scores for Each Primary Outcome. Results Are Presented as Odds Ratio (OR) and 95% Confidence Interval

	Odds ratio (95% confidence interval)	
	RAI-rev	*P*-value (two-sided)
Mortality	1.201 (1.142-1.246)	<0.001
Major complications	1.092 (1.063-1.121)	<0.001
Non-home discharge	1.153 (1.125-1.181)	<0.001
Wound complications	1.043 (0.994-1.095)	0.085
DVT	1.064 (0.991-1.144)	0.089
Readmission within 30 Days	1.040 (1.010-1.070)	0.008
Pulmonary complications	1.133 (1.084-1.185)	<0.001
Cardiac complications	1.130 (1.056-1.209)	<0.001
Infection	1.047 (1.016-1.080)	0.003
	**mFI-5**	***P*-value** (two-sided)
Mortality	1.103 (1.064-1.144)	<0.001
Major complications	1.002 (0.992-1.012)	0.738
Non-home discharge	1.055 (1.048-1.062)	<0.001
Wound complications	0.990 (0.975-1.005)	0.197
DVT	0.980 (0.955-1.005)	0.113
Readmission within 30 Days	0.997 (0.988-1.006)	0.492
Pulmonary complications	1.594 (1.209-2.101)	0.001
Cardiac complications	1.410 (0.905-2.195)	0.128
Infection	1.066 (0.929-1.223)	0.361

### Secondary Outcomes after Septic Revision

After controlling for age and sex, a multivariate analysis was conducted to assess associations of frailty indices with postoperative outcomes for each cohort (Figure 1, Tables 4 and 5). RAI-rev was shown to have significant associations with NHD, major complications, readmission within 30 days, pulmonary complications, cardiac complications, and postoperative infection while mFI-5 had significant associations with NHD, and pulmonary complications (Table 5). With increasing severity in RAI-rev, the association with NHD (Prefrail OR: 2.00; Frail OR: 2.81; Severely Frail OR: 9.79), postoperative infection (Prefrail OR: 1.45; Frail OR: 2.02; Severely Frail OR: 2.76), and cardiac complications (Prefrail OR: n/a; Frail OR: 1.06; Severely Frail OR: 3.33) significantly increased (Table 2).

### Predictive Value of Frailty Indices in Septic Revision

In order to determine the discriminatory accuracy of mFI-5 and RAI-Rev, AUC analysis (Figure 1, Table 6) was performed. AUC analysis revealed that RAI-Rev significantly outperformed mFI-5 for NHD (Cs: 0.670 vs 0.602, *P* < 0.001) and mortality (Cs: 0.795 vs 0.574, *P* < 0.001). There were no significant differences between RAI-Rev and mFI-5 for all other outcomes.

**Table 6.** Predictability of Each Frailty Index for Each Primary Outcome by Use of AUC Method and C-Statistics

Frailty index	RAI-rev	mFI-5	*P*-value (two-sided)
Mortality	0.795 [0.723-0.867]	0.574 [0.494-0.656]	<.001
Major complications	0.557 [0.488-0.631]	0.547 [0.471-0.622]	.582
Non-home discharge	0.670 [0.646-0.694]	0.602 [0.578-0.626]	<.001
Wound complications	0.503 [0.449-0.558]	0.525 [0.476-0.572]	.442
DVT	0.517 [0.469-0.565]	0.577 [0.522-0.631]	.196
Readmission within 30 Days	0.520 [0.488-0.552]	0.541 [0.509-0.573]	.202
Pulmonary complications	0.630 [0.581-0.678]	0.609 [0.560-0.661]	.558
Cardiac complications	0.644 [0.579-0.704]	0.590 [0.522-0.654]	.353
Infection	0.532 [0.481-0.582]	0.515 [0.465-0.567]	0.346

## Discussion

The goal of this study was to compare the discriminatory ability of mFI-5 and RAI-rev to predict postoperative outcomes in patients undergoing revision septic rTJA. Our findings demonstrate the positive association between frailty status and poor surgical outcomes overall, with the RAI-rev demonstrating superiority in comparison to the mFI-5, specifically, when predicting 30-day mortality and NHD. As a result, frailty can play a vital role in preoperative risk assessment, and the RAI-rev may provide that assessment most accurately. These indices can be tools to assist in educating patients and their family members about the risks involved with revision surgery, in addition to providing intraoperative and perioperative teams with an understanding of likely outcomes following these procedures to inform monitoring and prepare to address each risk in a timely, efficient manner.

While RAI-rev was originally developed to predict long-term mortality (6-12 months), it has also been proven to be an accurate predictor of 30-day mortality as well as NHD.^25,26^ Similar to other studies, RAI-rev, in our study, demonstrated the greatest difference in predictability compared to mFI-5 when examining mortality.^24,27^ Physiologic reserve is a difficult metric to quantify; however, the inclusion of RAI-rev’s variables such as cognitive deterioration, poor appetite, and activities of daily living allows for a more appropriate, frailty specific index.^19^ The incorporation of acute variables such as renal failure, functional status, age, and physiologic reserve, in addition to the comorbidities included in the mFI-5 may explain the superiority of the RAI-rev.^25,28^ The inclusion of these variables allows for a more accurate prediction of surgical risk in septic revision patients, as their acute functional status and physiologic reserve may predispose them to surgical site and systemic complications.^29^ Because of the difference in mortality rate following septic revisions of THAs/TKAs compared to that of aseptic revisions, utilizing the most predictive frailty indices in preoperative risk stratification is paramount.^10^

In addition to avoiding mortality following septic revision, minimizing the length of stay and optimizing disposition are paramount for shaping the postoperative course and improving cost overall. There have been multiple efforts to reduce the total costs associated with THAs and TKAs; however, the balance of optimal healthcare delivery and expenditures is difficult to manage.^30^ With this, discharge location can dramatically affect cost reduction and mitigate suboptimal healthcare delivery.^31-35^ In fact, Zeng et al found 90-day cost increases ranging from $6620 to $22,921 for discharges to a skilled nursing facility, respectively. Mitigating these costs may help lower the overall attributable cost for septic revision, as it has demonstrated to be twice as costly compared to aseptic revision arthroplasty in a recent systematic review.

Minimizing operative risk for major complications is also vital; Easterlin et al^36^ found an increased risk of major complications during primary knee arthroplasties beginning at age 70, and the risk increases as the patient ages. Comorbidities and delayed surgery are known risk factors that increase the likelihood of major complications, especially in hip fracture patients.^37,38^ In addition, the variables assessed by RAI-rev allow for the identification of frail patients at risk for postoperative major complications and account for additional risk factors like malnutrition.^39-43^ Understanding of the increased complication risk for each, unique patient undergoing septic rTJA can help surgeons schedule the procedure at capable facilities with the necessary means to accommodate risk factors associated with frailty such as malnutrition and non-home discharge.

An accurate preoperative risk stratification index is paramount. The association of frailty with negative postoperative outcomes highlights the role that the RAI-rev can play in allowing physicians to better determine the risk of each patient in addition to providing them with the opportunity to better educate the patient on the expectations of surgery.^25,44,45^ In addition, conferring these risks with the anesthesia and perioperative teams may allow for preparation in anticipation of likely complications encountered either intraoperatively or postoperatively to prevent more worrisome outcomes requiring ICU admission or mortality. Despite our advances in perioperative care, revision for PJI still carries a high perioperative risk and understanding this risk and being prepared to address these abnormalities in a timely manner is important to continue to improve our outcomes in this population.

### Limitations

The primary limitations of this study are those inherent to performing an analysis utilizing a large, multicenter database. The ACS-NSQIP database does not contain any pre-operative information such as cognitive status, nor does it include data exceeding 30 days post-operation; as a result, any complications arising after the 30-day period can reported, which could result in undocumented, negative postoperative outcomes that would be relevant to this study. Revision arthroplasty in any capacity has immediate concerns that should be monitored for in the 30-day period following surgery, but event-free perioperative periods do not always confer a successful outcome. Therefore, these findings may offer limited insight into the full outcome of patients undergoing revision arthroplasty and future studies should identify whether these events within 30-days are associated with long-term outcomes in this specific cohort. Additionally, certain preoperative demographic and patient-related variables associated with outcomes in previous studies like cognitive function, neurological status, previous spine instrumentation/lower extremity surgery, etc. Were not able to be pulled from the current database and may limit the generalizability of these findings. Likewise, the characteristics of the prosthetic joint infection (organism, number of CFUs, preoperative inflammatory labs, degree of infection, soft tissue/bony involvement) were not able to be reported and may limit the recommendations provided in this report. These patients reported to facilities within the United States. Their treatment modalities, cultural differences, and access to care may be different from that of non-US populations and severely limit the generalizability of these conclusions to centers outside of the US. The surgical approach, implant selection, alignment, specific radiographic characteristics, and detailed operative technique could not be fully elucidated from the NSQIP dataset and may impact the translation of these findings to clinical scenarios. Given the retrospective nature of this study, the results are unable to prove causation related to surgical intervention or frailty, and these results may differ when validation is attempted in prospective studies. In addition, ICD coding inaccuracies can result from insufficient documentation by healthcare providers, variations in clinical terminology, software glitches in electronic health record systems, and misunderstanding of the coding guidelines; these potential errors may affect our study’s outcomes.

## Conclusion

Frailty may have a distinct association with mortality and NHD in patients undergoing septic revision total hip and knee arthroplasty. The revised Risk Analysis Index may be more effective in assessing individual patient risk for the most worrisome outcomes following these procedures. The understanding of these outcomes is important in order to properly educate the patient and their family on their unique risk and provide insight into the necessary resources and surveillance needed to manage frail patients undergoing septic revision total joint arthroplasty.

## Supplemental Material

Supplemental material - Comparative Analysis of Frailty Indices on Complication Risk Following Septic Revision Total Hip and Knee Arthroplasty Supplemental material for Comparative Analysis of Frailty Indices on Complication Risk Following Septic Revision Total Hip and Knee Arthroplasty by Hannah Grimmett, BS, Arsalaan Sayyed, BS, Victor Koltenyuk, BA, Aruni Areti, BA, Hikmat R. Chmait, MS, Nithin Gupta, BS, Mitchell Gray, MD, William Young, MD, Tyler K. Williamson, DO, Chance Moore, MD, Frank A. Buttacavoli, MD in Geriatric Orthopaedic Surgery & Rehabilitation.

## Data Availability

Data utilized for this study is readily available upon request.*
