# Supplementary material for: Comparative Analysis of Frailty Indices on Complication Risk Following Septic Revision Total Hip and Knee Arthroplasty
Source: Geriatr Orthop Surg Rehabil. 2025 Oct 25;16:21514593251392692. doi: 10.1177/21514593251392692 (PMC12572601; doi:10.1177/21514593251392692)
Supplement: Supplemental material - Comparative Analysis of Frailty Indices on Complication Risk Following Septic Revision Total Hip and Knee Arthroplasty [file sj-pdf-1-gos-10.1177_21514593251392692.pdf]

| **mFI-5 Components** | **NSQIP Variable** | **Score** |
|---|---|---|
| Non-Independent Functional Status* | FNSTATUS2 | 1 |
| Diabetes mellitus with oral agents or insulin | DIABETES | 1 |
| COPD | HXCOPD | 1 |
| Hypertension Requiring Medication | HYPERMED | 1 |
| Congestive Heart Failure | HXCHF | 1 |
| The mFI-5 calculated using the 5 NSIP variables resulting in an index with a score of 0 = “least frail”, 1 = “prefrail”, 2 = “frail”, and ≥3 = “severely frail” | | |
| *Includes partial and complete dependence | | |

**Supplementary Table S1**: mFI-5 Calculation

| **RAI Components** | **NSQIP Variable** | **Score** |
|---|---|---|
| Sex | SEX | +3 if male |
| Age | AGE | + score with cancer (Supplementary Table S3) |
| Cancer Diagnosis | DISCANCER | Variable only relevant through age |
| Weight loss | WTLOSS | 4 |
| Renal Failure | DIALYSIS or RENAFAIL | 8 |
| Congestive heart failure | HXCHF | 5 |
| Poor appetite | WTLOSS | 4 |

| | | |
|---|---|---|
| Shortness of breath at rest | DYSPNEA | 3 |
| Residence other than independent living (transferred from nonhome or intermediate care unit) | TRANST | 1 |
| Cognitive deterioration (over past 3 months) | N/A | |
| Activities of daily living | FNSTATUS2 | Without cognitive decline |
| | | +14 = totally dependent |
| | | +7 = partially dependent |
| | | +0 = independent |
| **RAI-rev scoring ranges from Robust (RAI ≤15), Normal (RAI 16-25), Frail (26-35) and Severely frail (RAI ≥36)** | | |

**Supplementary Table S2**: RAI Calculation

| **Age (yr)** | **Score with Cancer** | **Score without Cancer** |
|---|---|---|
| ≤ 19 | 28 | 0 |
| 20-24 | 29 | 1 |
| 25-29 | 29 | 4 |
| 30-34 | 30 | 6 |
| 35-39 | 30 | 8 |
| 40-44 | 31 | 10 |
| 45-49 | 31 | 12 |
| 50-54 | 32 | 14 |
| 55-59 | 32 | 16 |
| 60-64 | 33 | 18 |
| 65-69 | 34 | 20 |
| 70-74 | 34 | 22 |

| | | |
|---|---|---|
| 75-79 | 35 | 24 |
| 80-84 | 35 | 26 |
| 85-89 | 36 | 28 |

**Supplementary Table S3**: RAI-rev Score of Age and Cancer Diagnosis
